# Oral metronomic vinorelbine combined with endocrine therapy in hormone receptor-positive HER2-negative breast cancer: SOLTI-1501 VENTANA window of opportunity trial

**DOI:** 10.1186/s13058-019-1195-z

**Published:** 2019-09-18

**Authors:** Barbara Adamo, Meritxell Bellet, Laia Paré, Tomás Pascual, Maria Vidal, José A. Pérez Fidalgo, Salvador Blanch, Noelia Martinez, Laura Murillo, Patricia Gómez-Pardo, Ana López-González, Kepa Amillano, Jordi Canes, Patricia Galván, Blanca González-Farré, Xavier González, Patricia Villagrasa, Eva Ciruelos, Aleix Prat

**Affiliations:** 1 Department of Medical Oncology, Hospital Clínic de Barcelona, Barcelona, Spain; 2 Translational Genomics and Targeted Therapeutics in Solid Tumors, IDIBAPS, Villarroel 170, 08035 Barcelona, Spain; 3 Vall d’Hebrón University Hospital/Vall d’Hebron Institute of Oncology (VHIO), Barcelona, Spain; 4 SOLTI Breast Cancer Research Group, Barcelona, Spain; 5 Hospital Clínico Universitario de Valencia/INCLIVA/CIBERONC, Valencia, Spain; 6 Fundación Instituto Valenciano de Oncología, Valencia, Spain; 7 Hospital Universitario Ramón y Cajal, Madrid, Spain; 8 Hospital Clínico Universitario Lozano Blesa, Zaragoza, Spain; 9 Complejo Asistencial Universitario de León, León, Spain; 10 University Hospital St. Joan de Reus, Reus, Spain; 11 Hospital Universitario 12 de Octubre, Madrid, Spain

**Keywords:** Breast cancer, Metronomic, Vinorelbine, Letrozole, Window of opportunity, Gene expression

## Abstract

**Background:**

The biological effect of oral metronomic vinorelbine (mVNB) alone or in combination with endocrine therapy in patients with hormone receptor-positive (HR+)/HER2-negative breast cancer has been scarcely addressed.

**Methods:**

Postmenopausal women with untreated stage I–III HR+/HER2-negative breast cancer were randomized (1:1:1) to receive 3 weeks of letrozole (LTZ) 2.5 mg/day, oral mVNB 50 mg 3 days/week, or the combination. The primary objective was to evaluate, within PAM50 Luminal A/B disease, if the anti-proliferative effect of LTZ+mVNB was superior to monotherapy. An anti-proliferative effect was defined as the mean relative decrease of the PAM50 11-gene proliferation score in combination arm vs. both monotherapy arms. Secondary objectives included the evaluation of a comprehensive panel of breast cancer-related genes and safety. An unplanned analysis of stromal tumor-infiltrating lymphocytes (sTILs) was also performed. PAM50 analyses were performed using the nCounter®-based Breast Cancer 360™ gene panel, which includes 752 genes and 32 signatures.

**Results:**

Sixty-one patients were randomized, and 54 paired samples (89%) were analyzed. The main patient characteristics were mean age of 67, mean tumor size of 1.7 cm, mean Ki67 of 14.3%, stage I (55.7%), and grades 1–2 (90%). Most baseline samples were PAM50 Luminal A (74.1%) or B (22.2%). The anti-proliferative effect of 3 weeks of LTZ+mVNB (− 73.2%) was superior to both monotherapy arms combined (− 49.9%; *p* = 0.001) and mVNB (− 19.1%; *p* < 0.001). The anti-proliferative effect of LTZ+mVNB (− 73.2%) was numerically higher compared to LTZ (− 65.7%) but did not reach statistical significance (*p* = 0.328). LTZ+mVNB induced high expression of immune-related genes and gene signatures, including CD8 T cell signature and PDL1 gene and low expression of ER-regulated genes (e.g., progesterone receptor) and cell cycle-related and DNA repair genes. In tumors with ≤ 10% sTILs at baseline, a statistically significant increase in sTILs was observed following LTZ (paired analysis *p* = 0.049) and LTZ+mVNB (*p* = 0.012). Grade 3 adverse events occurred in 3.4% of the cases.

**Conclusions:**

Short-term mVNB is well-tolerated and presents anti-proliferative activity alone and in combination with LTZ. The high expression of immune-related biological processes and sTILs observed with the combination opens the possibility of studying this combination with immunotherapy. Further investigation comparing these biological results with other metronomic schedules or drug combinations is warranted.

**Trial registration:**

NCT02802748, registered 16 June 2016.

**Supplementary information:**

**Supplementary information** accompanies this paper at 10.1186/s13058-019-1195-z.

## Background

In hormone receptor-positive and HER2-negative (HR+/HER2−) early breast cancer, adjuvant endocrine therapy for 5–10 years is recommended for all patients whereas multi-agent chemotherapy (mostly anthracycline/taxane-based) is recommended for patients with high-risk tumors [1]. However, despite treatment with adjuvant endocrine and multi-agent chemotherapy, patients with high-risk HR+/HER2− disease still have a substantial risk of relapsing [2–5]. A similar situation exists in advanced/metastatic HR+/HER2− disease, where the median overall survival does not exceed 30–35 months [6, 7]. Although recent randomized studies based on two different treatment strategies targeting the cell cycle showed exciting results [8–13], new therapies or treatment approaches are needed in order to improve the outcomes in HR+/HER2− disease.

Clinical development of metronomic therapy alone or in combination therapy has been scarce. Regarding breast cancer, different chemotherapy agents currently used have been evaluated within metronomic regimens, often combined with hormonal therapy, targeted agents such as trastuzumab or bevacizumab, or vaccines. Vinca alkaloids such as vinorelbine act mainly as mitotic spindle poisons which impair chromosomal segregation during mitosis [14, 15]. In common with other agents in this class, vinorelbine (25–30 mg/m^2^ iv on days 1 and 8 every 3 weeks) blocks cells at G2/M when present at concentrations close to the IC50 [14]. In a Luminal/HER2-negative breast cancer model (i.e., MCF7), vinorelbine at 2 nM induced apparent G1-phase accumulation as well as the induction of CDK inhibitor p21(WAF1/CIP1) protein and the dephosphorylated form of retinoblastoma protein [16]. In breast cancer, a wide range of phase II and III studies have now established the activity of vinorelbine alone, or in combination with other chemotherapeutic agents, in the treatment of early and advanced breast cancer [17]. More recently, studies have confirmed that metronomic oral vinorelbine can safely be administered at doses up to 50 mg three times a week [18, 19]. This strategy might not only affect the cell cycle but also target tumor angiogenesis and the immune system [20, 21].

Clinical trials in the preoperative setting collecting samples after 2 weeks of treatment have demonstrated the clinical validity of Ki67 as a predictor of benefit from endocrine treatment as well as a predictor of long-term survival outcome [22]. Therefore, a short-term non-therapeutic “window” studies might offer a clinical platform for rapid, efficient testing of anticancer agents and new combinations in breast cancer. Designed as a “window of opportunity” study, here we present the results of the SOLTI-1501 VENTANA trial, aiming to evaluate the anti-proliferative effect of oral metronomic vinorelbine (mVNB) alone or in combination with endocrine therapy in patients with untreated HR+/HER2− breast cancer.

## Methods

### Study design and participants

The VENTANA is a multicenter, window of opportunity, three-arm, randomized trial across ten hospitals in Spain. Female patients aged at least 18 years and postmenopausal were eligible if they had previously untreated, histologically confirmed stage I–IIIA invasive breast cancer, with primary tumors larger than 1 cm in diameter (as measured by ultrasound or MRI), clinical nodal status of 0–1, and HR-positive and HER2-negative according to ASCO/CAP guidelines [23, 24]. Patients also had to have an Eastern Cooperative Oncology Group (ECOG) performance status of 0–2 and adequate hematological counts and hepatic and renal function. Patients were excluded if they had multicentric tumors and received prior anti-cancer therapy. Detailed inclusion and exclusion criteria can be found in www.clinicaltrials.gov.

All patients provided written informed consent, and the protocol was approved by the Ethics Committees from all participating institutions and Spanish Health Authorities. The study was conducted in accordance with Good Clinical Practice principles, the Declaration of Helsinki, and all local regulations.

### Procedures

Patients were randomized (1:1:1) to receive letrozole (LTZ) 2.5 mg daily, oral mVNB 50 mg 3 days a week, or LTZ 2.5 mg daily and mVNB 50 mg three times a week during 3 weeks. Any adverse event and relationship to study medication were recorded and graded according to the National Cancer Institute Common Terminology Criteria for Adverse Events, version 4.0. After 3 weeks of treatment, patients underwent a surgery. Tumor samples were collected in less than 28 days before the therapy and after 3 weeks of treatment (within 5 days after the last dose). Following surgery, adjuvant treatment was as per investigator’s choice and local standards of care outside the scope of this protocol.

### Outcomes

Primary objective assessed if oral mVNB in combination with LTZ induce a superior anti-proliferative effect than either drug alone in patients with early breast cancer defined as luminal by PAM50. Proliferation was evaluated by measuring the mean expression of 11 proliferative-related genes contained in the PAM50 assay as previously described [25].

Secondary objectives included the assessment of the anti-proliferative of mVNB alone or in combination with endocrine therapy in patients with PAM50 Luminal A and Luminal B breast cancer. An unplanned analysis of stromal tumor-infiltrating lymphocytes (sTILs) and safety of the treatments was also analyzed.

### Gene expression analysis

A section of the formalin-fixed paraffin-embedded (FFPE) breast tissue was examined with hematoxylin and eosin staining to confirm the presence of invasive tumor cells and to determine the minimum tumor surface area. At least two 10-μm FFPE slides were used to purify total RNA using the High Pure FFPET RNA Isolation Kit (Roche, Indianapolis, IN, USA). Macrodissection was performed in baseline samples (when needed) to avoid contamination with normal breast tissue. A minimum of ~ 100 ng of total RNA was used to measure the expression of 752 breast cancer-related genes setting up the Breast 360™ Codeset using the nCounter platform (Nanostring Technologies, Seattle, WA, USA), which includes the 50 genes of the PAM50 subtype predictor and 702 additional genes that encompass 32 gene expression signatures (Additional file 1: Table S1). Data was normalized using 5 housekeeping genes and log2 transformed. Intrinsic molecular subtypes were identified using the research-based PAM50 predictor as previously described [26].

### Stromal tumor-infiltrating lymphocytes

sTIL determination was obtained from central review blinded from clinical-pathological and outcome data. Histopathological analysis of the proportion of sTILs was done in whole sections of tumor tissue stained with hematoxylin and eosin (H&E). sTILs were quantified according to the 2014 Guidelines developed by the International TILs Working Group [27].

### Ki67

Ki67 immunohistochemistry data was obtained from central review blinded from clinical-pathological and outcome data. Ki67 was assessed by immunohistochemistry using anti-Ki67 (30-9) rabbit monoclonal primary antibody (Ventana Medical System). In all samples, Ki67 interpretation criteria were done according to the latest international recommendations [28].

### Statistical analysis

This study is exploratory. The sample size chosen was not based on a formal statistical assumption since no prior window study has evaluated this combination and this biomarker (i.e., 11-gene proliferation signature). For the primary endpoint, the 11-gene proliferation score was used, which is calculated by obtaining the mean expression of 11 proliferation-related genes of the PAM50 assay (BIRC5, CCNB1, CDC20, CDCA1, CEP55, KNTC2, MKI67, PTTG1, RRM2, TYMS and UBE2C). All analyses regarding the proliferation score changes were performed on a per-protocol population, defined as all patients who completed 3 weeks of treatment and for whom tumor biopsy specimens were available for assessment of biologic response.

The association between two variables was evaluated using Student’s *t* test, Pearson’s *χ*^2^ test, or Fisher’s exact test. All statistical tests were two-sided and considered significant when *P* ≤ 0.05. To identify genes differentially expressed between paired baseline and surgical samples, a paired two-class significance analysis of microarrays (SAM) was used with a false discovery rate (FDR) ≤ 5%. All statistical analyses were performed using the R v3.2.3 software.

## Results

### Clinical-pathological characteristics

**Fig. 1:**
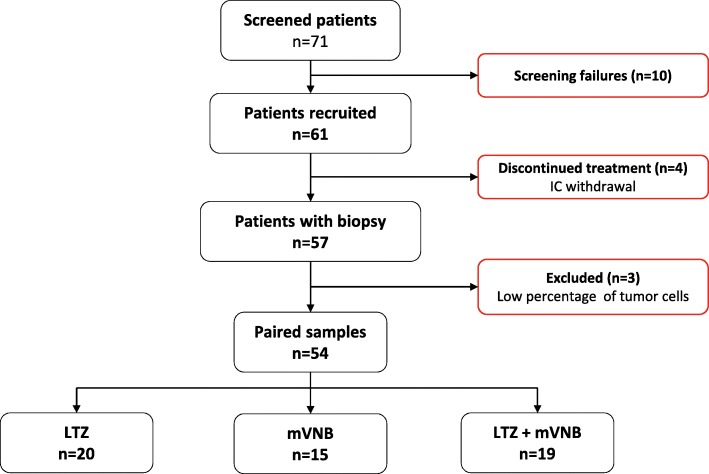
Flow chart of the SOLTI-1501 VENTANA study

From July 2016 to January 2018, 61 patients with newly diagnosed and untreated HR+/HER2− breast cancer were randomized across 10 centers in Spain. Patient characteristics are summarized in Table 1. Patients and tumor characteristics were balanced between the treatment groups. At baseline, the mean tumor size was 1.7 cm (range 0.8–3.5) and mean Ki67 was 14.3% (range 1–50%). Clinical stage I disease represented 55.7% (*n* = 34), and most patients had clinically node-negative (*n* = 58; 95.1%) and grade 1–2 (90%) disease. Finally, 4 patients withdrew consent, and the vast majority of patients (*n* = 57; 93.4%) completed the treatment as planned. Samples were obtained from 57 patients. Fifty-four paired samples (89%) were analyzed and reported here (Fig. 1).

**Table 1:** Patient demographics at baseline

	All patients, *N* = 61	Patients with paired tumor samples, *N* = 54
Mean age (range)	67 [53–86]	67 [53–86]
Tumor size (mean, range)	1.7 [0.8–3.5]	1.7 [0.8–3]
Clinical tumor stage		
T1	34 (55.7%)	30 (55.6%)
T2	27 (44.3%)	24 (44.4%)
Clinical lymph nodal status		
N0	58 (95.1%)	53 (98.1%)
N1	3 (4.9%)	1 (1.9%)
Histological grade		
G1	23 (37.7%)	21 (38.9%)
G2	32 (52.4%)	29 (53.7%)
G3	2 (3.3%)	2 (3.7%)
NA	4 (6.6%)	2 (3.7%)
ECOG		
0	54 (88.5%)	47 (87.0%)
1	7 (11.5%)	7 (13.0%)
Arms		
Letrozole	21 (34.4%)	20 (37.0%)
mVNB	20 (32.8%)	15 (27.8%)
Letrozole+mVNB	20 (32.8%)	19 (35.2%)

*NA* not available; *mVNB* metronomic vinorelbine

### Intrinsic subtype

**Fig. 2:**
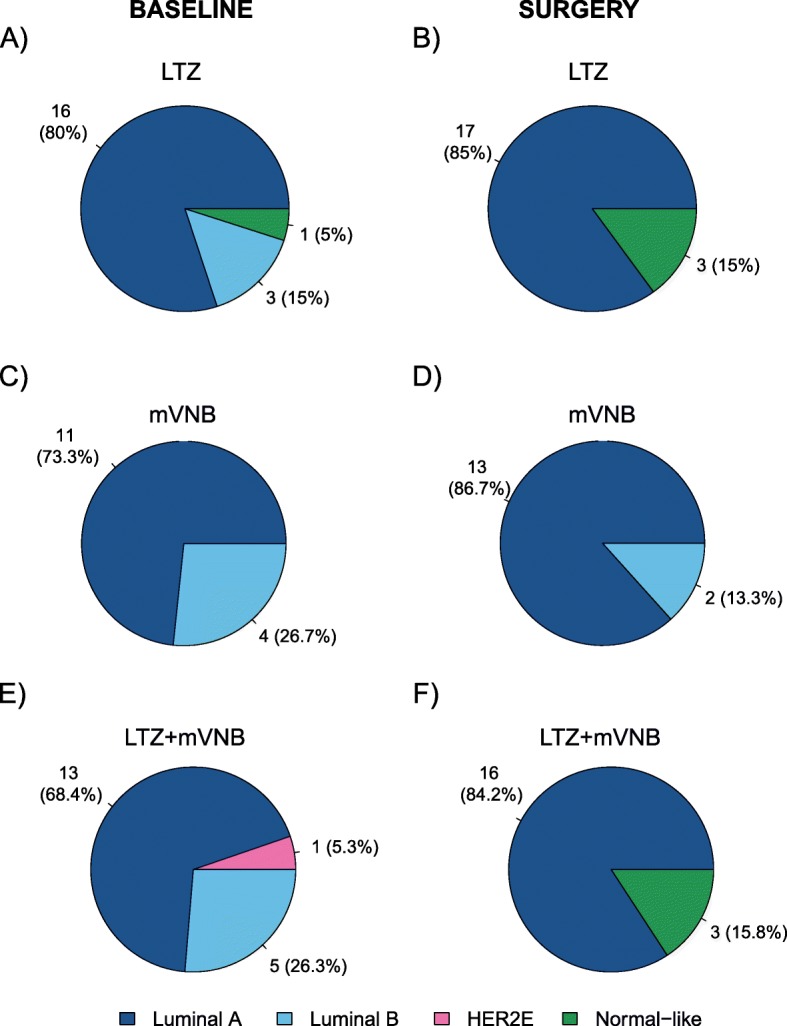
Distribution of the intrinsic subtypes before (**a**, **c**, **e**) and after treatment (**b**, **d**, **f**) in LTZ (**a**, **b**), mVNB-only (**c**, **d**), and combination (**e** and **f**) arms

At baseline, the distribution of the PAM50 intrinsic subtypes was as follows: Luminal A (*n* = 40, 74.1%), Luminal B (*n* = 12, 22.2%), HER2-enriched (*n* = 1, 1.9%), and Normal-like (*n* = 1, 1.9%). At surgery, the distribution of the PAM50 intrinsic subtypes was as follows: Luminal A (*n* = 46, 85.2%), Luminal B (*n* = 2, 3.7%), and Normal-like (*n* = 6, 11.1%). A significant decrease of Luminal B disease was observed at surgery compared to baseline (22.2% vs. 3.7%; *p* = 0.004). At baseline, Luminal A and Luminal B were identified in all arms (Fig. 2a, c, e), the only patient with HER2-enriched disease was identified in the combination arm (Fig. 2e). At surgery, Luminal B was identified only in the mVNB monotherapy arm (Fig. 2d). No statistically significant differences in intrinsic subtype were observed between baseline and surgery across the treatment arms (Fig. 2).

### Changes in the 11-gene proliferation score

**Fig. 3:**
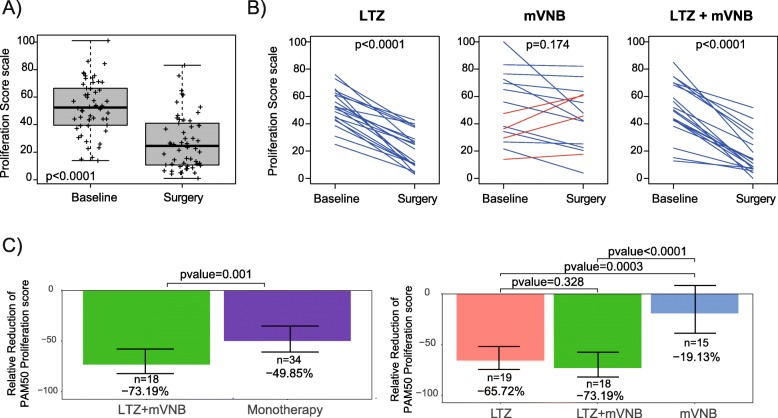
Relative reduction of PAM50 proliferation score in Luminal A/B disease. **a** The 11-gene proliferative score at baseline vs. surgery. **b** Changes in the proliferation score in each arm. **c** Primary results of the study: On the left, the anti-proliferative effect of the combination of mVNB and LTZ vs. both monotherapy arms combined. On the right, comparison of the anti-proliferative effect between each treatment arm. Error bars indicate 95% confidence interval

The 11-gene proliferative score in the baseline tumor samples was significantly higher than surgical samples (52.6 vs. 27.5; *p* < 0.0001) (Fig. 3a). Regarding the primary objective of the study, the anti-proliferative effect arm in luminal tumors of the combination of mVNB and LTZ was superior to both monotherapy arms combined (− 73.2% [95% IC − 82.9 to − 58.3%] vs. − 49.9% [95% IC − 61.3 to − 35.1%]; *p* = 0.001) (Fig. 3c). The anti-proliferative effect in the different arm in luminal tumors was as follows: in LTZ+mVNB arm, − 73.2% (95% IC − 82.9 to − 58.3%); in LTZ arm, − 65.7% (95% IC − 75.3 to − 52.5%); and in mVNB arm, − 19.1% (95% IC − 39.4 to 7.8%).

As a secondary objective, we compared the anti-proliferative effect between each treatment arm (Fig. 3c) and observed that both LTZ-containing arms showed a superior anti-proliferative effect compared to mVNB monotherapy (− 19.1%; *p* < 0.0001). No statistically significant difference was observed between the combination arm and LTZ-only arm (− 73.2% vs. − 65.7%; *p* = 0.328).

The correlation coefficient (*r*) between the 11-gene proliferation score and Ki67 by immunohistochemistry was strong (*r* = 0.76) (Additional file 2: Figure S1). Overall, a significant and profound decrease in the 11-gene proliferation score was observed in both LTZ arms (Fig. 3b). In the mVNB monotherapy arm, most tumors (73.3%) showed a decrease in proliferation (blue lines); however, this decrease was statistically non-significant; in addition, 4 of 15 patients showed an increase in proliferation in the mVNB monotherapy arm.

### Treatment safety

The most frequent adverse events were arthralgias, asthenia, diarrhea, hot flushes, and nausea and occurred in < 10% of the cases (Additional file 5: Table S2). Two cases (3.4%) of grade 3 adverse event, both in the mVNB-only arm, were observed after completing the 3-week treatment. One case was an acute pancreatitis in a patient with a history of cholelithiasis. The other case was an acute gastroenteritis. Overall, there were no discontinuations due to toxicity.

### Changes in selected genes and gene signatures

**Fig. 4:**
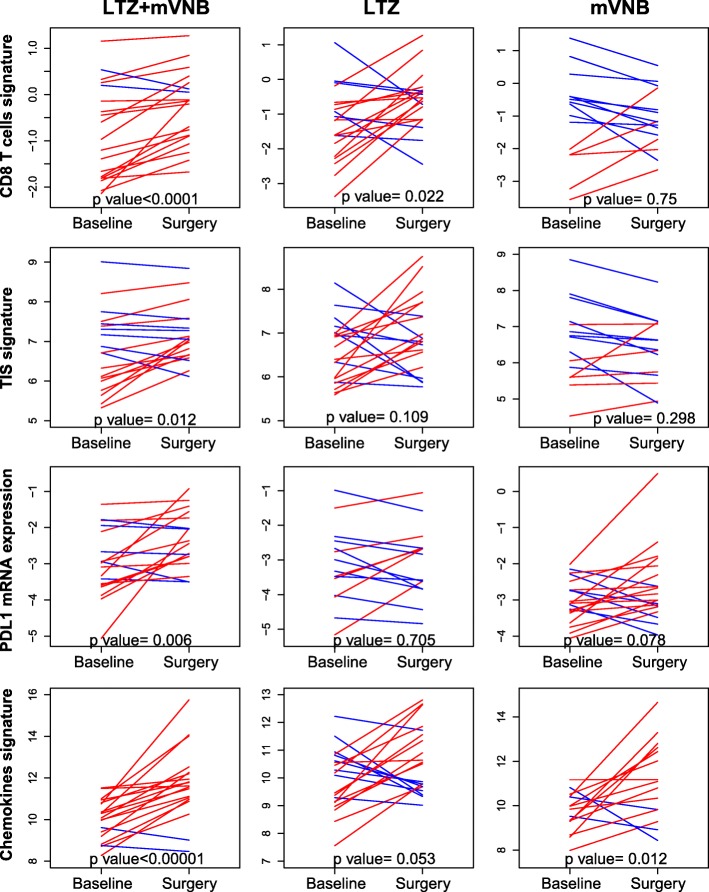
Biological changes induced by each treatment at week 3 compared to baseline of selected genes and gene signatures

**Fig. 5:**
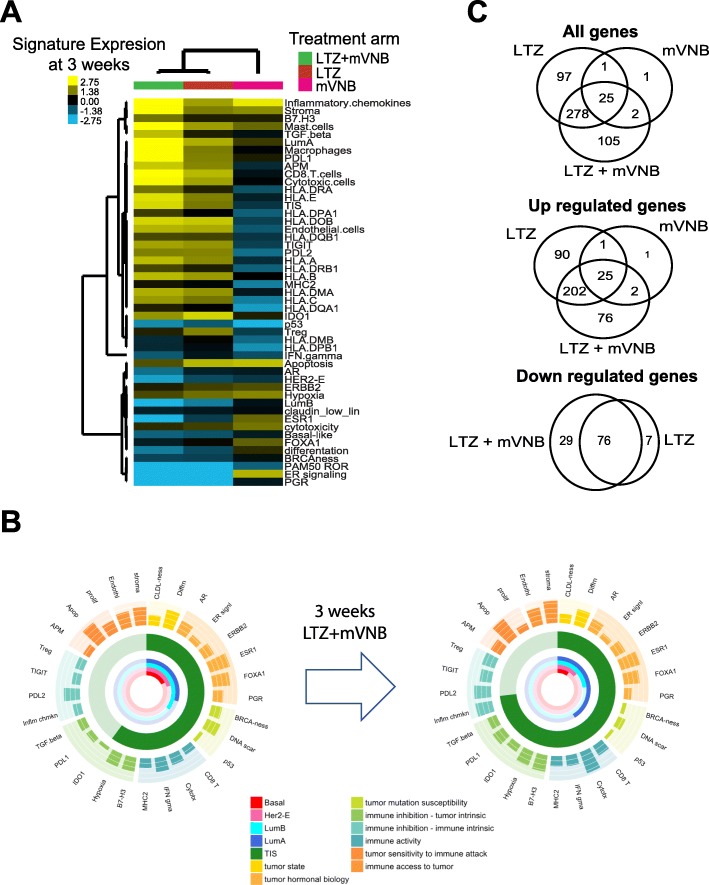
Global gene expression changes induced by each arm from baseline to surgery. **a** Heatmaps showing differential expression of 49 selected genes and gene signatures at 3 weeks in each arm. **b** 360° view summarizing the gene/signatures for the breast tumor microenvironment and immune response in LTZ+mVNB arm between baseline and surgery. **c** Venn diagram showing the overlap in the list of differentially expressed genes obtained in the before and after treatment across the comparisons of the three arms

To further identify the biological changes induced by each treatment, we explored the expression at week 3 compared to baseline of 49 selected genes and gene signatures tracking various biological processes such as estrogen-regulated genes, PAM50 risk of relapse (ROR) score, hypoxia, immune infiltration, ERBB2, estrogen receptor (i.e., ESR1), or immune checkpoint inhibitor PDL1 (Figs. 4 and 5a, b).

As expected, the expression of many other biological processes beyond proliferation changed during treatment. For example, both LTZ-containing arms induced a strong decrease of PAM50 ROR score, Luminal B signature, and ER-regulated genes, including ESR1 and progesterone receptor (PGR). On the contrary, both LTZ-containing arms, and specially the LTZ+mVNB arm, induced high expression of immune-related genes and gene signatures such as the Tumor Inflammation Signature (TIS) [29], the CD8 T cell signature, macrophages, and immune checkpoint inhibitors PDL1 and PDL2. Most of these changes in gene and gene signature expression were not identified in the mVNB-only arm, except for the high expression of the inflammatory chemokine signature (Figs. 4 and 5a). Compared to the other arms, the mVNB monotherapy induced a more differentiated luminal phenotype with increased expression of ESR1, estrogen-regulated gene signature, and FOXA1 (Fig. 5a).

### Immune infiltration

The previous results suggested that immune infiltration was enriched in post-treatment samples of the LTZ-containing arms, especially the combination arm. To further evaluate this, we identified the presence of sTILs before and after treatment. Overall, ≥ 10% of sTILs at 3 weeks were observed in 6.6% (1/15), 15% (2/20), and 26% (5/19) of the cases within the mVNB-only, LTZ-only, and combination arms, respectively (Additional file 3: Figure S2A). In tumors with ≤ 10% sTILs at baseline, a statistically significant increase in sTILs was observed following LTZ (*p* = 0.049) and VNB+LTZ (*p* = 0.012), but not in the mVNB-only arm (Additional file 3: Figure S2B). Thus, this pathology-based analysis confirms the previous gene signature-based results.

### Global gene expression changes

In this study, the expression of 752 genes and 32 breast cancer-related gene signatures were analyzed at both time points. Thus, we explored the changes in the expression of each individual gene in each treatment arm. After adjusting for multiple testing (FDR < 5%), the number of genes whose expression significantly changed in each arm was 410 (52.3%) in the LTZ+mVNB arm, 401 (51.2%) in the LTZ-only arm, and 29 (3.7%) in the mVNB-only arm (Fig. 5c and Additional file 4). As expected, a considerable overlap of genes (60%) occurred between both LTZ-containing arms (Fig. 5c).

Compared to the baseline samples, post-treatment samples in the combination arm showed higher expression of AP-1 transcription factor subunits FOS and JUN, inflammatory chemokines (e.g., CCL4, IL6, and CCL3L1), and stromal-related genes (e.g., CAV1). Concordant with this observation, the upregulated gene list in the post-treatment time point was found enriched with the following biological processes: inflammatory response, chemokine-mediated signaling pathway, chemotaxis, and adaptive immunity. On the contrary, post-treatment samples in the combination arm showed lower expression of cell cycle-related genes (e.g., AURKA and UBE2C), DNA repair (e.g., BRCA1, BRCA2 and RAD51), and microtubule (e.g., MAPT and KIF11).

Of the 410 genes found differentially expressed in the combination arm, 107 (26.1%) were not found in the LTZ-only differentially expressed gene list (Fig. 5c and Additional file 4). Within this gene list, 78 and 29 genes were upregulated and downregulated in post-treatment samples, respectively. Within the upregulated gene list, we identified the immune checkpoint inhibitor LAG3 and immune-related genes such as IL1B, CCR5, and CXCL8 (Additional file 4). Within the downregulated gene list, we identified the ESR1 and the proliferation-related genes CHEK2 and FANCF and others such as MTOR. From both gene lists, no significant enrichment for any particular biological process was identified.

In the LTZ-only arm, 98 (24.4%) differentially expressed genes were not found in the combination arm. Within this gene list, 91 and 7 genes were upregulated and downregulated in post-treatment samples, respectively (Fig. 5c and Additional file 4). However, no significant enrichment for any particular biological process was identified from both gene lists.

### Evaluation of the chemo-endocrine score

We have previously reported a PAM50-based chemo-endocrine score (CES) in HR+/HER2− early disease that predicts response to endocrine therapy vs. chemotherapy [30]. Here, we explored the association of CES (as a continuous variable and using the previously established cutoffs) with the biological response, measured as > 50% relative decrease in the 11-gene proliferation signature. In the LTZ-only arm, the response in the CES-high (high endocrine sensitive and low chemo-sensitive) and CES-med (low endocrine sensitive and high chemo-sensitive) groups was 72.7% (8/11) and 37.5% (3/8) of the patients, respectively. Of note, only one CES-low sample was identified in the LTZ-only arm. In the mVNB-containing arms, the response in the CES-high, CES-med, and CES-low groups was 50% (9/18), 45% (5/11), and 80% (4/5) of the patients, respectively. The interaction between CES (as a continuous variable) and treatment (LTZ vs. mVNB) was statistically significant (*p* = 0.039).

## Discussion

Prior studies have evaluated and studied the combination of vinorelbine and endocrine therapy. In the preclinical setting, synergistic activity between vinorelbine and hormone therapy has been observed [16]. In the clinical setting, a single-arm phase II clinical trial combining vinorelbine and tamoxifen in 38 evaluable patients as the first-line therapy, of whom 63% had positive and 29% unknown hormonal receptor status, the overall response rate was 66% and the complete response rate was 16%. No evidence of additive side effects was observed. Another single-arm clinical trial tested LTZ in combination with vinorelbine in postmenopausal women with metastatic breast cancer. This combination appeared to be an active regimen (overall response rate of 50%) with a favorable safety profile [31]. Interestingly, a phase III trial in patients with hormone-refractory prostate cancer, progression-free survival (PFS) was significantly prolonged when vinorelbine was combined with hormone therapy compared with hormone therapy alone [32].

From a biological perspective, the secondary and exploratory results of the VENTANA trial suggest that adding mVNB to LTZ might lead to the expression of specific biological features. Several observations support this hypothesis. On one hand, according to gene expression data, mVNB monotherapy induces a biological profile consistent with high endocrine dependency, with upregulation of ESR1, FOXA1, and the ER-regulated gene signature and downregulation of the 11-gene proliferation signature. On the other hand, although the two LTZ-containing arms show a very similar biological profile after treatment, the LTZ+mVNB arm has a more pronounced and consistent upregulation of immune-related genes and gene signatures, an increase in sTILs, and a more pronounced and consistent downregulation of proliferation and cell-cycle-related genes. Thus, it is plausible that mVNB has the ability to differentiate the tumor cells into a slightly more estrogen-dependent state, and in this context, LTZ is more effective. Further studies are needed to confirm this hypothesis. However, other treatments that inhibit the cell cycle such as PI3K inhibitors have shown to increase estrogen dependency of the tumor cell in the preclinical setting [33]. Another hypothesis is that mVNR affects cells within a tumor with the highest proliferation, leaving untreated those that are not sensitive to chemotherapy but are likely highly sensitive to letrozole.

A low proliferation in response to preoperative endocrine therapy predicts for a good long-term outcome, whereas high levels have been associated with an increased risk of breast cancer recurrence [34]. In our study, the anti-proliferative effect arm in luminal tumors of the combination of mVNB and LTZ was superior than that in the monotherapy arms. Ki67 detected by immunohistochemistry is currently the most used marker to estimate tumor cell proliferation. The Ki67 score measured at 2 weeks in IMPACT [35], at 16 weeks in P024 [36], and at 2 to 4 weeks in ACOSOG Z1031 [37] was predictive of relapse-free survival in multivariate analysis, whereas the pretreatment Ki67 was not. Utilizing the long-term outcomes of the patients from P024, Ellis et al. found that pathologic stage (tumor size and nodal status), in addition to the levels of the protein Ki67, and Allred ER score measured on the surgical specimen were independently associated with both relapse-free survival and breast cancer-specific survival [38]. The preoperative endocrine prognostic index (PEPI score) was developed based on these findings and validated in the outcome data from the IMPACT and ACOSOG Z1031 trials. The ongoing randomized phase III ALTERNATE clinical trial (NCT01953588) in women with HR+/HER2− early breast cancer is testing a biomarker-driven treatment strategy based on Ki67 values following 4 and 12 weeks of neoadjuvant hormonotherapy and the PEPI score to identify women at low risk of disease recurrence.

HR+/HER2− breast cancer is generally considered non-immunogenic [39, 40]. In agreement with previous studies, only 9.3% (5/54) of tumor samples in our study showed > 5% of stromal sTILs. However, an interesting result is the increase in immune-related genes and signatures after 3 weeks of treatment in both LTZ-containing arms, especially with the LTZ+mVNB combination. This observation is consistent with prior studies. For example, 6 months of letrozole alone or in combination with metronomic cyclophosphamide was able to reduce the presence of intra-tumoral FOXP3+ Tregs in 83 elderly breast cancer patients [41]. In another study, an increased expression of genes related to inflammatory processes, with enrichment of those promoting T cell anergy, was observed after 3 months in responders to neoadjuvant anastrozole [42]. The immune effects of anastrozole have also been described in rat models, with increased levels of proinflammatory cytokines and suppression of Treg differentiation induced by this drug. A combination approach with pembrolizumab, an anti-PD1 antibody, in association with anastrozole, exemestane, or letrozole (in the ER-positive cohort of the study) is being evaluated in patients with ER-positive metastatic breast cancer (NCT02648477), and neoadjuvant durvalumab, an anti-PDL1 antibody, is being explored in a phase II trial (ULTIMATE, NCT02997995) in combination with exemestane in patients whose tumor is found inflamed (i.e., > 10% CD8+ T cells) after 3 weeks of exemestane with tremelimumab, an anti-CTLA4 antibody.

Metronomic chemotherapy schedule seems to have not only a direct cytotoxicity on cancer cells but also an effect on the tumor microenvironment by modulating immune response [43–47]. However, in our study, the mVNR alone did not increase immune metrics as much as letrozole in monotherapy. On one hand, the dose of mVNR might be too low to induce this effect. On the other hand, the duration of mVNR might be too short. Indeed, studies with metronomic schedules that support the effect on the immune system have a longer duration.

The randomized design of this window of opportunity trial allowed us to explore the ability of our previously described PAM50-based CES signature to predict response to chemotherapy vs. endocrine therapy. As expected, CES showed the inverse pattern of association as previously reported, where high CES values are associated with endocrine sensitivity and chemo-resistance and the low values are associated with endocrine resistance and chemo-sensitivity. Despite the low number of patients in each arm, the interaction test between CES and treatment for their association with a response was statistically significant. This result gives us a strong rationale to test, in an upcoming collaboration, the predictive value of CES to adjuvant chemotherapy in the Danish Breast Cancer Cooperative Group 77B clinical trial, which randomized 1072 premenopausal women to no systematic treatment (control), levamisole, cyclophosphamide, or cyclophosphamide-methotrexate-fluorouracil arms [48, 49].

Our study has limitations worth noting. First, the sample size chosen was not based on a formal statistical assumption since no prior window study had evaluated this combination and this biomarker (i.e., 11-gene proliferation signature). We do note, however, that the observed magnitude of difference in our study for the primary objective (i.e., 23.3%) and the given sample size has a statistical power of 83.6%. Considering the meaning of a post hoc analysis as a prospective measure, this power means that a new trial testing the same hypothesis with the same sample size and biomarker will have a probability of 83.6% of ending up with a *p* value of less than 5%. Second, whether longer duration of treatment might induce different results is unknown. Third, no claims regarding clinical benefit can be made. However, our results suggest that adding endocrine therapy in patients with advanced HR+/HER2− disease that is treated with mVNB might not be detrimental and might actually be of potential benefit. The results of a prior study support this hypothesis. Bottini and colleagues [50] completed a neoadjuvant randomized phase II trial where they combined letrozole with low-dose oral cyclophosphamide for 6 months. The investigators observed an overall response rate of 71.9% in the 57 patients randomly assigned to receive primary letrozole and 87.7% in the 57 patients randomly assigned to receive letrozole plus cyclophosphamide. In addition, there was a significantly greater suppression of Ki67 expression in the letrozole/cyclophosphamide-treated group than in the letrozole-treated group. Fourth, we focused our molecular analysis on a set of 752 genes and 32 gene signatures. Whether other biological processes might be occurring and are being missed by our study is unknown.

## Conclusions

To conclude, short-term mVNB in combination with LTZ presents high anti-proliferative activity and is well-tolerated compared to monotherapy. However, anti-proliferative activity does not seem to be higher than LTZ alone. Further investigation comparing these biological results with other metronomic schedules or drug combinations is warranted. In particular, the high expression of immune-related biological processes and sTILs observed with the combination opens the possibility of studying this combination with immunotherapy. Further investigation comparing these biological results with other metronomic schedules or drug combinations is warranted.

## Supplementary information

**Additional file 1: Table S1.** Breast Cancer 360 Biological signatures.

**Additional file 2: Figure S1.** Correlation coefficient (r) between the 11-gene proliferation score and Ki67 by immunohistochemistry.

**Additional file 3: Figure S2.** Changes in sTILs from baseline to surgery. A) stromal TILs across the treatment arms. B) In tumors with ≤10% sTILs at baseline.

**Additional file 4.** Raw gene expression, normalized gene expression, clinical data and results of the SAM analysis.

**Additional file 5: Table S2.** Summary of the most frequent adverse events (AE).

## Data Availability

The datasets generated and analyzed during this study are available from the corresponding authors on reasonable request.

## Abbreviations

LTZ: Letrozole
mVNB: Metronomic vinorelbine
HR+: Hormone receptor-positive
sTILs: Stromal tumor-infiltrating lymphocytes
HR+/HER2−: Hormone receptor-positive and HER2-negative
ER: Estrogen receptor
PFS: Progression-free survival
CDK: Cyclin-dependent kinase
ECOG: Eastern Cooperative Oncology Group
FFPE: Formalin-fixed paraffin-embedded
SAM: Significance analysis of microarrays
FDR: False discovery rate
ROR: Risk of relapse
PGR: Progesterone receptor
TIS: Tumor Inflammation Signature
CES: Chemo-endocrine score
