# Supplementary material for: Oral metronomic vinorelbine combined with endocrine therapy in hormone receptor-positive HER2-negative breast cancer: SOLTI-1501 VENTANA window of opportunity trial
Source: Breast Cancer Res. 2019 Sep 18;21:108. doi: 10.1186/s13058-019-1195-z (PMC6751874; doi:10.1186/s13058-019-1195-z)
Supplement: Supplementary file 1 — Additional file 1: Table S1. Breast Cancer 360 Biological signatures. [file 13058_2019_1195_MOESM1_ESM.docx]

## Table S1. Breast Cancer 360 Biological signatures

| **PROCESSES** | **SIGNATURES** |
| --- | --- |
| Breast cancer subtyping | PAM50 Molecular subtyping |
|  | Claudin-Low subtyping |
|  | Triple Negative Breast cancer subtyping |
| Breast cancer receptor signaling | ESR1 gene expression |
|  | PGR gene expression |
|  | ERBB2 gene expression |
|  | Estrogen receptor signaling |
| Tumor responsiveness | Antigen processing machinery |
|  | HRD |
|  | BRCA |
|  | P53 |
| Tumor regulation | Apoptosis |
|  | Proliferation |
|  | Differentiation |
|  | FOXA1 gene expression |
| Inhibitory tumor mechanisms | IDO1 gene expression |
|  | PD-L1 gene expression |
| Stromal factors | Endothelial cells |
|  | Stromal abundance |
| Inhibitory metabolism | Hypoxia |
| Anti-tumor immune activity | Tumor Inflammation Signature (TIS) |
|  | Cytotoxicity |
|  | Interferon gamma signaling |
|  | MHC class II antigen presentation |
| Inhibitory immune signaling | Inflammatory chemokines |
|  | PD-1 gene expression |
|  | TIGIT gene expression |
| Immune cell population abundance | Cytotoxic cell abundance |
|  | CD8+ T Cell abundacne |
|  | Macrophage abundance |
|  | Mast cell abundance |
|  | Treg abundance |
