# Supplementary material for: Oral metronomic vinorelbine combined with endocrine therapy in hormone receptor-positive HER2-negative breast cancer: SOLTI-1501 VENTANA window of opportunity trial
Source: Breast Cancer Res. 2019 Sep 18;21:108. doi: 10.1186/s13058-019-1195-z (PMC6751874; doi:10.1186/s13058-019-1195-z)
Supplement: Supplementary file 2 — Additional file 2: Figure S1. Correlation coefficient (r) between the 11-gene proliferation score and Ki67 by immunohistochemistry. [file 13058_2019_1195_MOESM2_ESM.docx]

**Fig. S1.** Correlation coefficient (r) between the 11-gene proliferation score and Ki-67 by immunohistochemistry
