# Supplementary material for: Oral metronomic vinorelbine combined with endocrine therapy in hormone receptor-positive HER2-negative breast cancer: SOLTI-1501 VENTANA window of opportunity trial
Source: Breast Cancer Res. 2019 Sep 18;21:108. doi: 10.1186/s13058-019-1195-z (PMC6751874; doi:10.1186/s13058-019-1195-z)

**Fig. S2.** Changes in sTILs from baseline to surgery. A) stromal TILs across the treatment arms. B) In tumors with ≤10% sTILs at baseline.


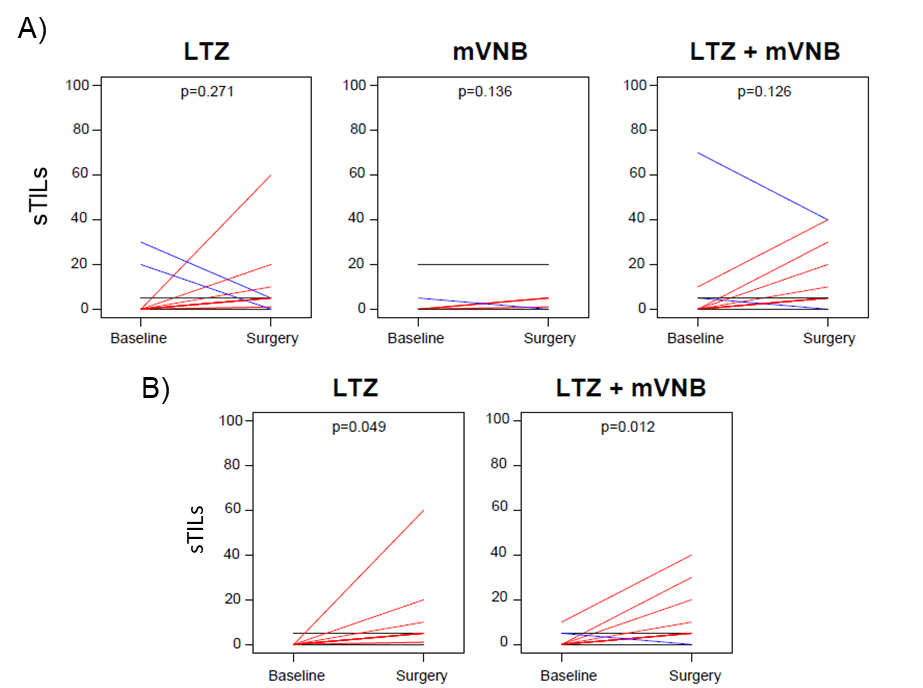

Supplement: Supplementary file 3 — Additional file 3: Figure S2. Changes in sTILs from baseline to surgery. A) stromal TILs across the treatment arms. B) In tumors with ≤10% sTILs at baseline. [file 13058_2019_1195_MOESM3_ESM.docx]
