# Supplementary material for: Oral metronomic vinorelbine combined with endocrine therapy in hormone receptor-positive HER2-negative breast cancer: SOLTI-1501 VENTANA window of opportunity trial
Source: Breast Cancer Res. 2019 Sep 18;21:108. doi: 10.1186/s13058-019-1195-z (PMC6751874; doi:10.1186/s13058-019-1195-z)
Supplement: Supplementary file 5 — Additional file 5: Table S2. Summary of the most frequent adverse events (AE). [file 13058_2019_1195_MOESM5_ESM.docx]

## Table S2. Summary of the most frequent adverse events (AE).

| **Safety Population n=58** | **LTZ**  **(N=21)** | | **LTZ + mVNB**  **(N=19)** | | **mVNB**  **(N=18)** | |
| --- | --- | --- | --- | --- | --- | --- |
|  | **N** | **%** | **N** | **%** | **N** | **%** |
| **Arthralgia** | 2 | 9.5 | 2 | 10.5 | 0 | 0.0 |
| **Asthenia** | 1 | 4.8 | 0 | 0 | 3 | 16.7 |
| **Diarrhea** | 0 | 0 | 1 | 5.3 | 2 | 11.1 |
| **Hot flushes** | 2 | 9.5 | 0 | 0 | 1 | 5.6 |
| **Nausea** | 0 | 0 | 3 | 15.8 | 2 | 11.1 |
